# Remarks on Medical Legislation

**Published:** 1850-03

**Authors:** Erial McArthur

**Affiliations:** Chicago, Ill.


### ARTICLE V.

*Remarks on the Propriety of Legislative Enactments for the purpose of Elevating the Standard of Medicine and Medical Ethics in the State of Illinois.*—By ERIAL MCARTHUR, M.D., of Chicago, Ill.

This, no doubt, will be considered by many, as a very delicate question, and one fraught with difficulty, both in its conception and progress, as regards its adaptation to the wants of an honorable and philanthropic Profession, and the best interests of society.

The question has generally been considered in the light of protection to the "regular," (Allopathist,) Physician. As an humble member of this school of medicine, I disclaim any such desire; and furthermore, I believe that a large share of our brethren, if the truth was known, would also disclaim any such hope, wish, or desire.

I purpose, therefore, in this communicaton, to set forth what I conceive to be necessary to meet the real wants of the Profession, in this point of view; and whatever tends to elevate and enlighten any class of citizens, will have the ultimate effect of benefiting all with whom they associate. This may be considered an axiom, from which none will dissent.

But in order to ascertain what Legislative Enactments would be calculated to meet the wants of the citizens of this commonwealth, it may be well to briefly inquire, what is the present condition of the Profession,--or more properly, what are the attainments, qualifications, and usual practices of those who set themselves up as being qualified to administer to the afflicted, the sick and the dying, in this, to them, a time of need?

There are several classes of men---or in other words, men of different qualifications, who are either *justly* entitled to the appellation of Doctor of Medicine, or who *assume* to themselves this title of distinction and importance.

1st. Those who have complied with the usual course of study required at our Medical Colleges, to wit: Three years time devoted to studies connected with the foundation principles of the Profession in the office of a respectable physician, such as Theoretical and Practical Anatomy, do. Chemistry, Pathology, Physiology, Hygiene, Surgery, Materia Medica, Obstetricy, Medical Jurisprudence; and last, though not least, the Institutes and the Practice of Medicine. It may be well to observe in this connection, that those who pursue this course to enter the portals of the Profession, also enjoy more or less Hospital and Clinic instruction, attend two full courses of lectures, (of sixteen weeks esch,) at a medical college where competent and practical teachers are supplied, pass a thorough and critical examination in all the branches above mentioned, and then, and not till then, receive the degree of Doctor of Medicine. It is but justice to the more enlightened part of our Profession, to state, that the above limited course of instruction is considered as too meager to meet the responsibilities that devolve upon the Physician, or the increased wants of society.

2d. There are a multitude of men scattered abroad through our State, and elsewhere, who have studied one, two, or even three years; some with practitioners, others without any teacher, without any clinics, and with few books, and them very likely obsolete and discarded; some who have perhaps attended part of a course, or even a full course of lectures; others there are who obtain two, three or more medical books, and half a dozen articles of medicine, a pair of turnkeys and a painted tin---then migrate to some interior or back settlement; study a little, and practice as occasion offers, and alas! for the cause of humanity, these are called Doctors. All of these may be considered as *belonging* to the regular bred, or *hanging on* to the skirts of the regularly educated and time-honored Profession.

3d. Then we find a few scattered abroad, who have *nominally complied* with the requirements of the Medical Schools, and who have *finally passed* the ordeal, and having their Diplomas in their pockets, try to gain a livelihood in the *regular line* of the Profession, but who are too superficial in honesty, intellect or acquirements, to succeed. And lo! and behold!---they have found a more excellent way. The curious are excited; and the credulity of the credulous is raised to its highest pitch. These may be considered regular quacks. It may be said without fear of successful contradiction, that not one in a thousand, who has pursued the regular course of instruction and succeeded in business, has ever left our ranks.

4th. In this class we have Hydropathists, Homœopathists, *Root-opathists*, *Herb-opathists*, *Nerve-opathists*, and a multitude of other "pathists" too numerous to mention, of such a heterogeneous mass as utterly to defy description. A few of these, (a very few) perhaps have been educated somewhat, but a very large share have not education sufficient to teach a common school.

And yet each and every grade and variety find some adherents and patrons, and that even in what is called civilized and enlightened society. Those mentioned in this enumeration have no regular term of pupilage or requirements *established.* Some of them may spend a week, some a month, and possibly there may be some of them who study a a year, before they assume to be qualified to mitigate the sufferings, soothe the woes, and heal the multiplied diseases which flesh is heir to.

I will now state briefly what I deem to be necessary in the way of Statute Law, to meet this condition of things, existing in this State, to wit: That a law be passed to the following effect:

"That no person shall practice Medicine and Surgery in the State, unless he be a Graduate of a Chartered Medical College in which the requirements are at least three years' study in the office of a respectable and qualified Physician, and the attendance upon two full courses of lectures, examination, &c.; or that the individual shall have passed a full and satisfactory examination before a Board of State Censors, created and appointed for that purpose, in each and all of the following branches of Medical Education, viz: Anatomy,

Physiology, Pathology, Chemistry, Materia Medica, Surgery, Obstetricy, and the Institutes of Medicine."

A knowledge of these several branches is indispensable to an enlightened practice of medicine, whatever system of practice a man may embrace, or whatever views he may entertain. And the plan recently recommended of reducing the fees at the Medical Institutions, if carried out and generally practised, (as it probably will be) will greatly increase the facilities for acquiring a sound and thorough knowledge of the principles and ethics connected with the scientific discharge of the duties of the profession, and that without any increased expenditure on the part of the aspirants. With a knowledge which a thorough and practical course of instruction in these branches would afford, a man would be qualified to discriminate between the right and the wrong. It would surely be a mark of weakness to shun the light.

The course and time of instruction and examination being fixed, we ask for no further protection. Let the matter, as to whether a physician shall be orthodox or not, rest with the individual practitioner and the intelligence of society. But we do ask that society be protected from the multitude of mountebanks and visionary pretenders, who quarter themselves upon society like leeches, and draw from thence the treasures, and not unfrequently the life's blood. Our laws recognize in the other professions, a strict conformity to certain rules and *regulations*. Are the consequences depending upon the practice of Medicine and Surgery of so little importance as to require no protection from Statute Law? Justice requires that a standard of Professional attainment should be defined by law; and unless doctors will comply with and obey just and liberal laws, let them be dealt with as are other transgressors of order and propriety. We do ask for qualifications on the part of those, who shall assume the prerogatives of our profession, that the community may be protected from fraud and imposition. We ask for no special prerogatives for this school, or the other, of medicine. We believe that no enlightened and judicious man can dissent from the above conclusion after a full examination of the subject.—There may be a discrepancy of opinion in relation to the best method of correcting existing evils.

It may not be amiss to mention some circumstances connected with this view of the subject in this city, and of most of which we are personally cognizant:

One man in this place, who assumes the prerogatives of the profession, was a hatter by trade in a neighboring State. He bought a small book purporting to be a medical book—settled in the interior of this State, and all at once was a doctor. After practising in the country a few years he came to this city, and the report was industriously circulated that he had been a 'regular bred,' but had found the more excellent way. Having accidentally met with him in a friend's house, whose son had fractured the radius and ulna of the right arm, he unblushingly declared, before several respectable witnesses, "that No. 6, (hot drops) would *cure* the broken bone, so that the boy could carry a pail of water in ten days. In order to make his statement more conclusive, he stated what he was pleased to call a fact, viz: "that in a certain place a consultation was held by the scien*tific* doctors in the case of an individual having a sore on the arm, and that they decided that the arm must be amputated, but the patient would not submit, and sent for a *Botanic Physician*, who applied No. 6, and the bad portion of the sore *slowed* (sloughed) off, and the arm got well.

Another man here with the cognomen of Dr., was a wheat buyer in the streets, and all at once, as if by magic, he became a professional man. Another was a teamster, etc., and he too suddenly became a Dr. And yet another was a whitewasher, and practised his art in the interior of the State, who, on moving into this city, assumed the dignity and title of the profession. And these individuals, as strange as it may seem, receive a liberal support in this community.

These circumstances are mentioned, not because they are deemed any thing new, or as being peculiar to this place. It is probable that almost every practitioner in the State is conversant with a multitude of quite similar circumstances; and hence the necessity of action to do away with these evils. In a democratic country, there can be no reasonable objection to individuals having pursued these several callings, becoming professional men, provided they qualify themselves for the discharge of these new duties, by a proper discipline of the mind, scholastic attainments, and a preliminary pupilage in the express duties they assume to perform. In the professions of Divinity and Law, a certain preliminary course of study is requisite before a man is *permitted* to engage in the specific duties of these professions.

Shall the profession, whose mission it is to mitigate and soothe the sufferings of humanity in all its varied relations to civilized life,—shall it be left open to the merciless and hard-hearted wretch, who engages in the discharge of solemn, momentous and intricate duties,—of the nature and tendency of which he knows comparatively little and cares less, and that without any regard to consequences,—whether it be a lifetime of suffering or a speedy death, merely that his sordid nature may be gratified in the anticipated acquisition of property and influence, which he fears would not be secured as a reward of humble toil and labor? If we do not look to this matter, and act upon it as a Profession, we need not expect any thing to be done. The community at large is too much engrossed with its own pursuits. It tacitly leaves these matters, which pertain to the Medical Profession, to the action of its own members, to regulate and govern. If we take honorable and elevated action upon this subject, there need be no fears but that an enlightened community will cheerfully and promptly sustain us.

It is thought that the present is a favorable time to have this subject presented for reflection and consideration. And we

[illegible] no better method to reach the profession at large, in this State, than through the medium of the Journal in most general circulation in the State.

The Convention called at Springfield on the first Tuesday of June next, to form a State Medical Association, (or State Medical Society,) will afford a favorable time to have the subject called up, and the opinion of the Profession generally obtained in regard to it. If it should be thought expedient and proper that Legislative action should be had, next winter would be a convenient and proper time to have the subject brought before the Legislature of the State for definite action. The importance to the Profession of the different practitioners of the State, or as many of them as convenient, meeting in convention and forming a State Medical Society, and conferring upon matters of interest to the Profession, cannot be over estimated.

We hope a liberal spirit will prompt many to make the necessary sacrifice of time and money, and come together for mutual action and benefit, and that the interests of the Profession shall be elevated thereby, and the cause of humanity ultimately promoted.

